# Supplementary material for: Comparison of Commercial Fish Proteins’ Chemical and Sensory Properties for Human Consumption
Source: Foods. 2023 Feb 24;12(5):966. doi: 10.3390/foods12050966 (PMC10000493; doi:10.3390/foods12050966)

Supplementary materials

Table S1. Sensory attributes, their descriptions and reference samples.

Attribute	Description	Reference sample (anchored value)
**Sedimentation**	The amount of sediment at the bottom the sample cup without stirring.	Pictures at the endpoints of the scale (0 and 10)
**Color saturation**	The saturation of the color	Three-point *(#ABABAB - #EEE8D2 - #FAE25B)* colour gradient from grey (0) to white (5) to yellow (10) covering the whole scale
**Cloudiness**	The visibility of the sample cups bottom from the top.	-
**Odor intensity**	The overall intensity of the odor	-
**Fishy odor**	The intensity of fishy or fish trimmings odor	Cooked herring *(Clupea harengus)* fillet (Salmonfarm Oy, Finland) (8)
**Brothy odor**	The intensity of brothy, meat jelly like odor	2% (w/w) BaseTaste yeast extract solution (Lihel Oy, Finland) (8)
**Seaweed/sea odor**	The intensity of seaweed like odor	2% (w/w) Bladderwrack (Wild Irish Seaweeds Ltd, Ireland) water solution (5)
**Particle distinctiveness**	The amount of sand like particles on the surface of the mouth	-
**Oily mouthfeel**	The amount of oily mouthfeel on surface of the oral cavity	-
**Flavor intensity**	The overall intensity of the flavor	Cooked herring *(Clupea harengus)* fillet (Salmonfarm Oy, Finland) (9)
**Seaweed/sea flavor**	The intensity of a seaweed flavor	-
**Metallic flavor**	The intensity of the metallic flavor	0,001 M $FeSO_4$ solutions aftertaste (9)
**Fishy flavor**	The intensity of fishy flavor	Cooked herring *(Clupea harengus)* fillet (Salmonfarm Oy, Finland) (8)
**Bitter flavor**	The intensity of bitter flavor	0,5% (w/w) caffeine water solution (6)
**Umami flavor**	The intensity of umami flavor	0,6% (w/w) natrium glutamate (50%), inosine 5-monophosphate (IMP) (25%) and guanosine 5-monophosphate (GMP) (25%) water solution (7)

Figure S1. Spider plot of the mean values of the sensory evaluation.

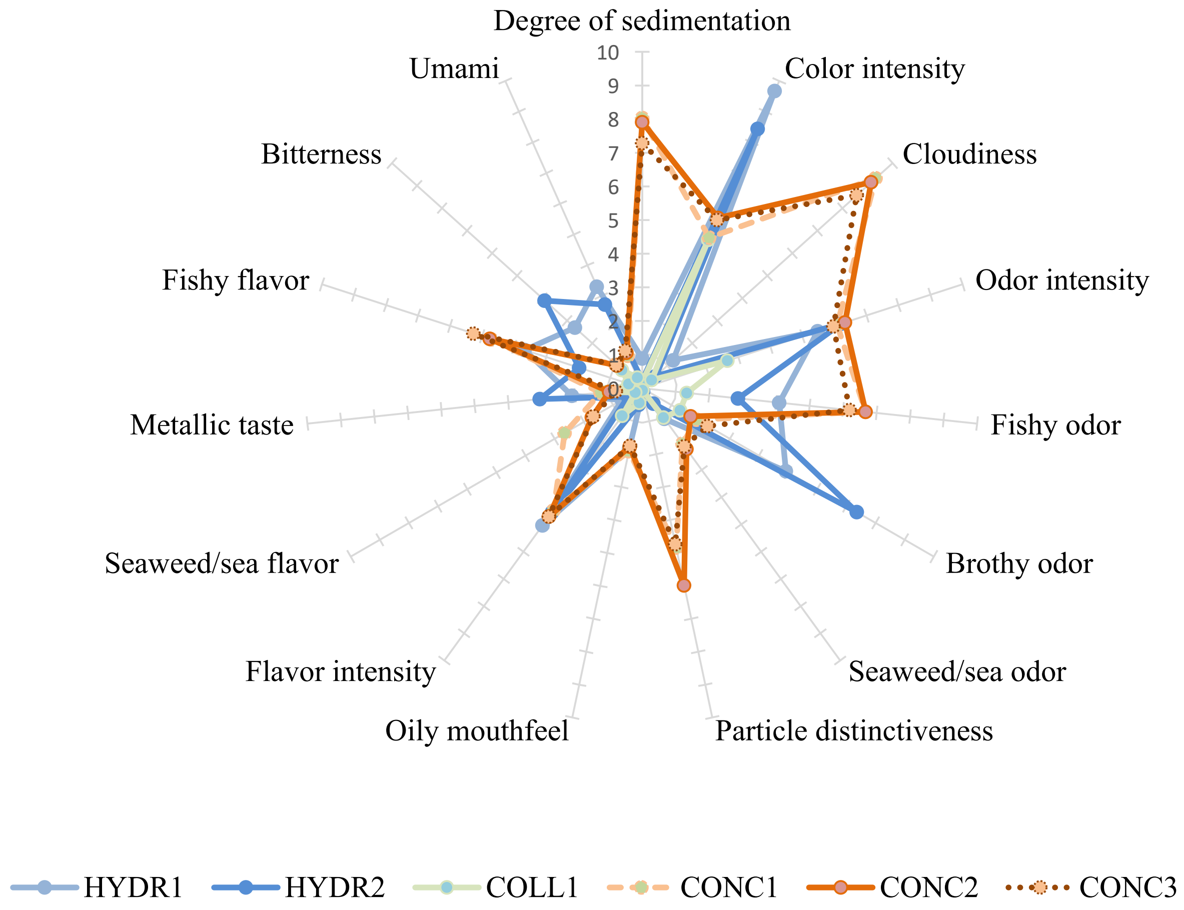

Table S2. The detection frequencies (DF) and odor descriptions of compounds detected with GC-MS-O (VF-Wax column) from commercial fish proteins.

#	Odor description	Volatile compound	RI	Identification	DF (%)		
					CONC3	HYDR1	HYDR2
1	Fishy, fatty	Trimethylamine	< 600	RI, O, MS	100	100	100
2	Cabbage, musty	Methanethiol	700	RI, O, MS	75	50	100
3	Sour bread	3-Methylbutanal	929	RI, O, MS	50	75	ND[1]
4	Sweet, butter, toffee	2,3-Butanedione	986	RI, O, MS	75	75	50
5	Glue, medicinal	Unknown 1	1026		75	ND	ND
6	Unclear	2,3-Pentanedione	1073	RI, MS	50	ND	ND
7	Grass	Hexanal	1103	RI, O, MS	50	25	25
8	Coffee, solvent	Unknown 2	1115		ND	75	50
9	Medicinal, burnt	2-Heptanone	1202	RI, O, MS	25	50	50
10	Fishy, pungent	(Z)-4-Heptenal	1263	RI, O, MS	100	75	75
11	Metallic	2-Octanone	1307	RI, O, MS	ND	50	35
12	Citrus, metallic	Octanal	1310	RI, O, MS	ND	25	25
13	Mushroom	1-Octen-3-one	1324	RI, O	100	75	ND
14	Mushroom, meat, broth	Hydroxyacetone	1337	RI, O, MS	ND	ND	100
15	Dog food, leather	Unknown 3	1358		ND	75	ND
16	Popcorn, basmati rice	2-Acetyl-1-pyrroline	1375	RI, O	100	100	50
17	Floral, raspberry, green	Unknown 4	1394		ND	ND	100
18	Metallic, iron	Unknown 5	1396		75	ND	ND
19	Musty	2,3,5-Trimethylpyrazine	1435	RI, O, MS	50	ND	ND
20	Soy sauce, green	3-Isopropyl-2-metoxypyrazine	1438	RI, O	ND	100	ND
21	Ink, musty, burnt	Unknown 6	1446		ND	ND	75
22	Boiled potato	Methional	1492	RI, O	100	75	75
23	Burnt, blackcurrant	Unknown 7	1543		ND	ND	100
24	Sour, pungent	1-Octanol	1559	RI, O, MS	50	ND	75
25	Musty, green, pungent	Unknown 8	1595		ND	75	ND
26	Pungent	2-Undecanone	1621	RI, O, MS	75	ND	ND
27	Berry	Unknown 9	1646		50	50	ND

28	Musty, sulfuric	2-Methylbutanoic acid	1687	RI, O, MS	ND	75	50
29	Meat broth	2-Dodecanone	1727	RI, O, MS	ND	75	ND
30	Liquorice, cloying	Unknown 10	1757		75	75	ND
31	Sweet, cotton candy	Unknown 11	2074		50	75	100
32	Rhubarb, acidic	Unknown 12	2084		ND	ND	50
33	Solvent, ink	Unknown 13	> 2186		50	ND	ND

CONC3 = fish protein concentrate, HYDR1-2 = Fish protein hydrolysates

[1] ND = Not detected

Figure S2. Partial least regression correlation loading of odor-active compounds and odor and flavor sensory properties of commercial fish proteins.

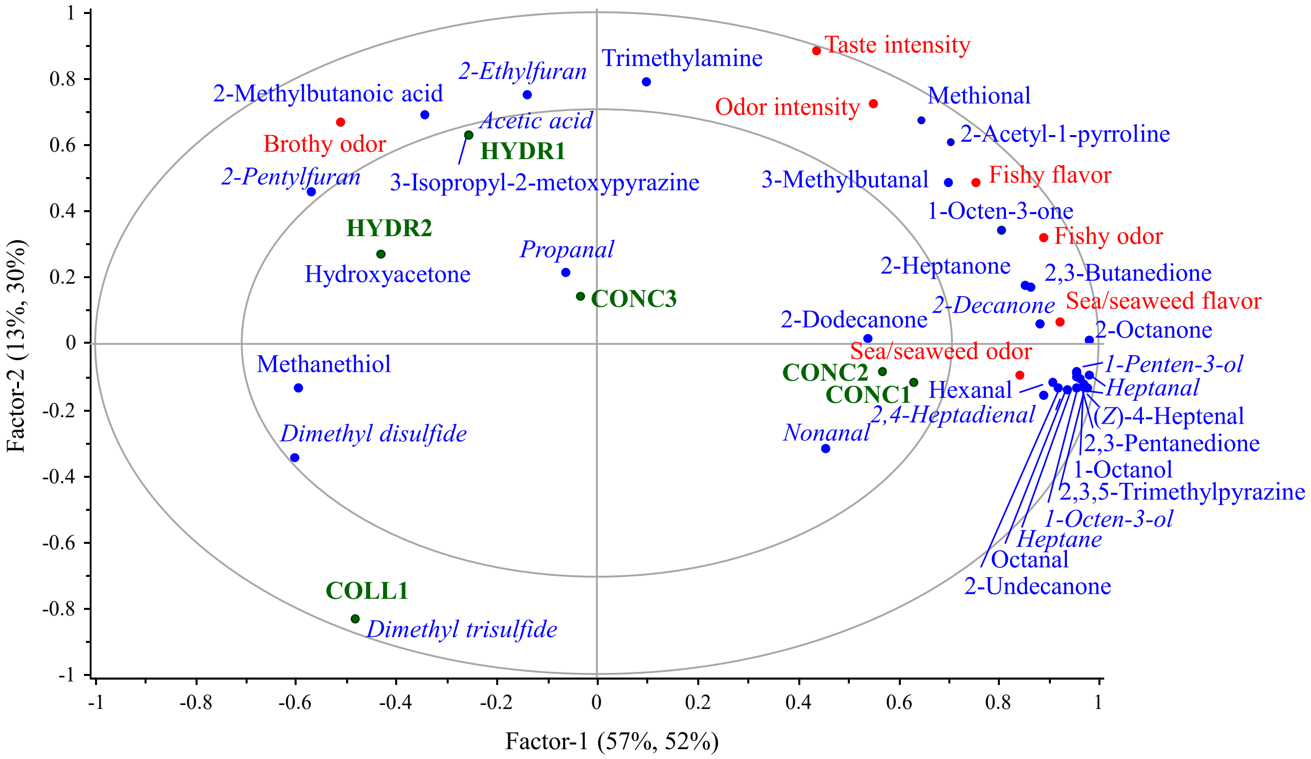

Supplement: Supplementary file 1 [file foods-12-00966-s001.zip › foods-2222798-supplementary.pdf]
